# An Observational Study on Patients With COVID-19 Infection Admitted to the Intensive Care Unit With Respect to Their Vaccination Status

**DOI:** 10.7759/cureus.37159

**Published:** 2023-04-05

**Authors:** Sounak Ghosh, Saswati Sinha, Mohua Bhattacharyya, Chandan Biswas, Subhash Todi, Rupak Kundu

**Affiliations:** 1 Department of Internal Medicine, Advanced Medicare and Research Institute (AMRI) Hospitals, Kolkata, IND; 2 Department of Critical Care Medicine, Advanced Medicare and Research Institute (AMRI) Hospitals, Kolkata, IND; 3 Department of Anesthesiology and Intensive Care Medicine, Yeovil Hospital NHS Foundation Trust, Yeovil, GBR

**Keywords:** sars-cov-2, vaccination, pandemic, intensive care unit, covid-19

## Abstract

Background

SARS-CoV-2 (COVID-19) created unprecedented recurrent waves of pandemic globally. Apart from COVID-19-appropriate behavior, vaccinating the population was proposed to be the most effective measure to control these outbreaks. However, the outcomes of vaccinated patients admitted to the intensive care unit (ICU) and their comparison with unvaccinated counterparts, especially in developing countries, have not been extensively studied.

Materials and methods

Our study examined consecutive patients with positive RT-PCR for COVID-19 admitted to the ICU from August 1, 2021, to July 31, 2022. Prior vaccination status and its relation to demographics, disease severity, mortality, and length of stay were analyzed.

Results

Among 436 patients admitted to the ICU, 76 (15.4%) were unvaccinated and 369 (84.6%) were vaccinated against COVID-19. Vaccinated patients were significantly older and hypertensive, and had comparatively less severity of illness than unvaccinated patients. Crude ICU and hospital mortality were significantly lower among vaccinated patients than unvaccinated patients (15.2% versus 25.4% and 16% versus 22.3%, respectively; P<0.05). Furthermore, risk-adjusted multivariate analysis demonstrated a strong but statistically nonsignificant inverse association between vaccination status and ICU mortality (odds ratio (OR)=0.540, 95% confidence interval (CI)=0.290-1.006, P=0.052).

Conclusion

In severe COVID-19-infected patients who required admission to the ICU, the majority were vaccinated. However, the severity of illness and hospital mortality was significantly lower among vaccinated patients with breakthrough infections.

## Introduction

SARS-CoV-2 (COVID-19) created recurrent waves of pandemic globally, resulting in high morbidity and mortality. Vaccinating the global population and COVID-appropriate behavior were proposed as the two most important approaches for controlling the pandemic [1]. The vaccination drive in India started on January 16, 2021, and since then, multiple vaccine types have been used with variable efficacy [2]. The majority of studies on the effectiveness of vaccination are community-based in the general population or conducted in a specific subset of the population, e.g., healthcare workers [3]. The effectiveness of vaccines and the timing at which the protective effect starts has been variably reported in the published literature [4]. For two-dose vaccines, vaccines only give partial protection after the first dose, and the second dose increases that protection [5-7]. Most studies have reported that COVID-19 vaccination, either partial or full, decreases the rate of symptomatic infection and also decreases the severity in symptomatic patients with less need for hospitalization when compared to the unvaccinated populations [8].

As reported by Grasselli et al. [9] prior to the vaccination drive, intensive care unit (ICU) mortality was noted to be 48.5% among 1,775 patients admitted to the ICU with severe COVID-19 infection [9]. However, studies on vaccinated patients who developed a symptomatic infection due to COVID-19 requiring hospitalization are limited. A study from Israel reported significantly reduced disease severity and mortality following vaccination [10]. Recently, a study was conducted in India among vaccinated individuals with COVID-19 infection requiring ICU admission, which showed a marked decrease in mortality among patients who received two doses of a vaccine [11]. However, this study excluded unvaccinated patients. To study this knowledge gap, we analyzed a cohort of critically ill patients with a confirmed diagnosis of COVID-19 by RT-PCR and determined their prior vaccination status. We studied the demographics, biochemical parameters, severity of illness, and outcome of infection in vaccinated patients (partial or full) and compared this to unvaccinated COVID-19 patients admitted during the same time period. In contrast to community-based vaccination studies, our study cohort consists of only critically ill patients admitted to the ICU.

## Materials and methods

This retrospective observational study was conducted in a 30-bed ICU of a tertiary care hospital in eastern India. The ICU is under the round-the-clock supervision of senior intensivists, and all interventional procedures were done by anesthesiologists or skilled residents under the supervision of anesthesiologists or intensivists. The data of patients admitted in the ICU with confirmed COVID-19 by RT-PCR between August 1, 2021, and July 31, 2022, were prospectively collected by data entry operators as a part of the standard protocol, and the data was stored in a secured electronic database. For the purpose of this study, we retrieved the data from medical records and excluded patients who had ages <18 years, had ICU stays of <24 hours, had unknown vaccination status, and were discharged against medical advice.

The enrolled patients were divided into two groups, vaccinated and unvaccinated, based on vaccination status. The vaccination statuses of the patients were determined by clinical records and post-discharge telephone surveys in patients with missing data on vaccination in clinical records. Data on dates of vaccination was collected. Moreover, demographic parameters, comorbidities, and baseline biochemistries of the included patients were collected from the clinical records. Baseline Acute Physiology and Chronic Health Evaluation (APACHE) IV and Sequential Organ Failure Assessment (SOFA) scores were calculated for all patients. The primary outcome of this study was ICU mortality. The secondary outcomes were hospital mortality, ICU and hospital length of stay, and the number of days on ventilation.

Statistical analyses

After confirming the final study population, variables were tested for normal distribution. The baseline characteristics of the participants were analyzed using mean±standard deviation (SD) for continuous variables and percentages for categorical variables. We used an independent sample t-test for continuous variables and a chi-square test for categorical variables for analyzing the differences between patients in different vaccination groups. Furthermore, univariate and multivariate logistic regression analyses were done to test for any significant independent relationship between vaccination status and primary outcome. We used the Statistical Package for the Social Sciences (SPSS) version 22 (IBM SPSS Statistics, Armonk, NY, USA) for all data analyses, and a two-tailed P value of <0.05 has been considered significant.

## Results

**Figure 1:**
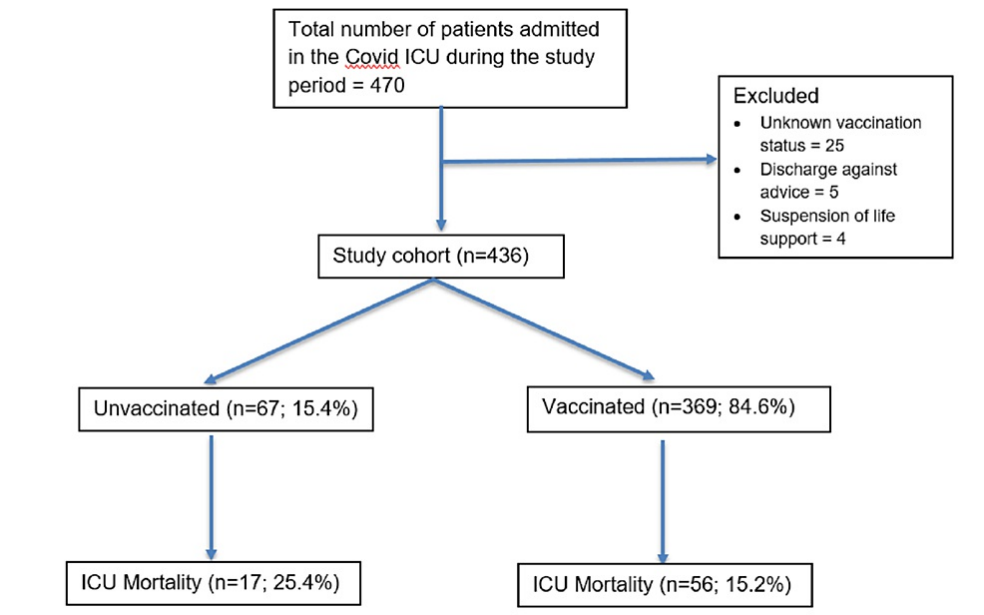
CONSORT flow diagram COVID-19: coronavirus disease 2019, ICU: intensive care unit

During the study period, a total of 504 patients were admitted to the ICU with confirmed COVID-19 infection. Out of these, 436 patients met the inclusion and exclusion criteria and constituted the study cohort (Figure 1). The mean age of the cohort was 70.65±13.07 years, and 275 (63.1%) were males. The majority of the patients (n=369, 84.6%) were vaccinated. Hypertension (68.3%) was the most prevalent comorbidity, followed by diabetes mellitus (58.25%) and chronic airway disease (20.64%). ICU and hospital mortality were 16.7% (n=73) and 17% (n=74), respectively (Table 1). Mortality was significantly higher among elderly patients (75.27 versus 69.71 years, P<0.001). Baseline serum biochemistry showed significantly altered renal function, low albumin, and raised transaminases among patients who died (Table 2). Among patients with malignancy/immunosuppression, both ICU and hospital mortality were similar (n=9, 19.6%).

**Table 1:** Characteristics of the study cohort Data are presented as numbers (percentages) or means±standard deviations. CKD: chronic kidney disease, COPD: chronic obstructive pulmonary disease, ICU: intensive care unit

Parameters	Frequencies
Mean age, years	70.65±13.07
Male	275 (63.1)
Female	161 (36.9)
Hypertension	298 (68.3)
Diabetes	254 (58.25)
CKD	46 (10.6)
COPD	90 (20.64)
Malignancy/immunosuppression	39 (8.94)
No vaccine	67 (15.4)
Vaccinated	369 (84.6)
ICU mortality	73 (16.7)
Hospital mortality	74 (17)
Mean ICU stay, days	4.12±2.26
Mean hospital stay, days	8.36±6.34

**Table 2:** Differences in demographics and biochemical parameters across survivor and non-survivor groups *P<0.05 Data are presented as numbers (percentages) or means±standard deviations. WBC: white blood corpuscle, HB: hemoglobin, AST: alanine transaminase, AST: aspartate transaminase, CRP: C-reactive protein, GGT: gamma-glutamyl transferase

Parameters	Survivors (n=362, 83%)	Non survivors (n=74, 17%)	P value
Age, years	69.71±13.36	75.27±10.41	<0.001*
Male	222 (61.3)	53 (71.6)	0.060
Female	140 (38.7)	21 (28.4)	
Vaccinated	309 (85.6)	59 (79.8)	0.046*
Hematocrit	34.38±6.20	33.48±6.56	0.368
WBC	9.3±6.1	9.6±7.8	0.770
HB, gm/dL	11.23±2.16	10.72±2	0.065
Platelet	211.8±172.11	186.6±72	0.258
Creatinine, mg/dL	1.37±1.18	2.08±1.86	<0.001*
Urea, mg/dL	43.93±3.92	82.48±70.26	<0.001*
Albumin, mg/dL	3.63±0.5	3.08±0.68	<0.001*
Bilirubin, mg/dL	0.76±1.31	1.03±2.52	0.220
AST	74.85±206.64	383.25±1608.49	0.001*
ALT	59.98±175.37	169.78±676.63	0.013*
GGT	61±94.01	89.57±152.81	0.059
Mechanical ventilation	19 (5.2)	38 (51.4)	<0.001*

Vaccinated patients admitted to the ICU with severe COVID-19 illness were predominantly elderly and hypertensive (Table 3). In contrast, a significant majority of unvaccinated patients had an underlying hematologic or solid organ malignancy than their vaccinated counterparts (30 (44.77%) versus 9 (2.43%); P=0.001). Apart from mildly low serum albumin, no differences in baseline biochemistry were observed between the vaccinated and unvaccinated groups (Table 4). The APACHE IV score was significantly lower among vaccinated patients (Table 5). Importantly, there was significantly reduced ICU (25.4% versus 15.2%; P=0.034) and hospital (22.4% versus 16%; P=0.044) mortality in this group. However, no differences were found in ICU and hospital length of stay. Likewise, the need for mechanical ventilation was significantly higher for unvaccinated patients than for vaccinated patients (19.45% versus 11.6%; P=0.047). The use of noninvasive ventilation and high-flow nasal oxygen was similar. The pO2:FIO2 (P:F) ratio was found to be raised among the vaccinated population (Table 6).

**Table 3:** Differences in demographics among unvaccinated and vaccinated groups *P<0.05 Data are presented as numbers (percentages) or means±standard deviations. CKD: chronic kidney disease, COPD: chronic obstructive pulmonary disease

Parameters	No vaccine (n=67, 15.4%)	Vaccinated (n=369, 84.6%)	P value
Age, years	70.05±12.77	73.94±14.22	0.025*
Male	43 (64.2)	232 (62.9)	0.477
Female	24 (35.8)	137 (37.1)	
Hypertension	130 (29.81)	168 (38.53)	0.041*
Diabetes	120 (27.52)	134 (30.73)	0.189
CKD	30 (6.88)	16 (3.66)	0.413
COPD	38 (8.20)	52 (11.92)	0.113
Malignancy/immunosuppression	30 (44.77)	9 (2.43)	0.001*

**Table 4:** Differences in baseline biochemistry among unvaccinated and vaccinated groups *P<0.05 Data are presented as numbers (percentages) or means±standard deviations. WBC: white blood corpuscle, HB: hemoglobin, AST: alanine transaminase, AST: aspartate transaminase, CRP: C-reactive protein, GGT: gamma-glutamyl transferase

Parameters	No vaccine (n=67, 15.4%)	Vaccinated (n=369, 84.6%)	P value
Glucose, mg/dL	192.88±95.69	180.76±89.22	0.381
Hematocrit	34.48±5.59	34.19±6.38	0.771
WBC	9.6±8.4	9.3±6.05	0.790
HB, g/dL	11.23±1.75	11.13±2.21	0.725
Platelet	228.3±214.2	202.8±145.8	0.301
Creatinine, mg/dL	1.43±1.30	1.50±1.35	0.712
Urea, mg/dL	48.03±34.75	51.32±46.73	0.609
Albumin, mg/dL	3.34±0.64	3.57±0.62	0.013*
Bilirubin, mg/dL	0.58±0.38	0.85±1.73	0.247
AST	45.98±30.09	143.78±76.39	0.327
ALT	27.52±21.44	88.61±35.32	0.185
GGT	48.87±56.31	69.68±41.76	0.190
CRP, mg/dL	69.59±69.21	90.79±76.72	0.197
Procalcitonin	1.13±0.5	2.9±1.3	0.484
pH	7.41±0.11	7.29±0.03	0.512

**Table 5:** Outcome differences among unvaccinated and vaccinated groups *P<0.05 Data are presented as numbers (percentages) or means±standard deviations. APACHE: Acute Physiology and Chronic Health Evaluation, SOFA: Sequential Organ Failure Assessment, ICU: intensive care unit

Parameters	No vaccine (n=67, 15.4%)	Vaccinated (n=369, 84.6%)	P value
APACHE IV	53.03±27.71	44.74±28.13	0.028*
SOFA	4.71±2.63	4.13±3	0.141
ICU mortality	17 (25.4%)	56 (15.2)	0.034*
Hospital mortality	15 (22.4%)	59 (16)	0.044*
ICU stay, days	4.34±3.90	4.08±2.33	0.692
Hospital stay, days	8.96±6.66	8.25±6.28	0.418

**Table 6:** Differences in organ support among unvaccinated and vaccinated groups The P:F ratio equals the arterial pO2 (“P”) from the ABG divided by the FIO2 (“F”). *P<0.05 Data are presented as numbers (percentages) or means±standard deviations. P: pO2, F: FIO2, ABG: arterial blood gas

Parameters	No vaccine (n=67, 15.4%)	Vaccinated (n=369, 84.6%)	P value
Mechanical ventilation	13 (19.4)	44 (11.9)	0.047*
Days on mechanical ventilation	8±5.62	8.86±6.83	0.680
Noninvasive ventilation	8 (11.9)	53 (14.4)	0.380
High-flow nasal oxygen	21 (31.3)	112 (30.4)	0.488
P:F ratio	163.64±125.86	231.06±187.95	0.033*

In univariate analysis, apart from age, APACHE IV, SOFA score, and mechanical ventilation, vaccination status was also found to be statistically significantly associated with reduced mortality (odds ratio (OR)=0.526, 95% confidence interval (CI)=0.283-0.978, P=0.042) (Table 7). Multivariate analysis adjusted for APACHE IV showed the trend toward the protective effect of vaccination, but the result was not statistically significant (OR=0.540, 95% CI=0.290-1.006, P=0.052) (Table 8).

**Table 7:** Univariate logistic regression of risk factors and ICU mortality *P<0.05 ICU: intensive care unit, APACHE: Acute Physiology and Chronic Health Evaluation, SOFA: sequential organ failure assessment, OR: odds ratio, CI: confidence interval

Parameters	OR	95% CI	P value
Vaccination	0.526	0.283-0.978	0.042*
Age	1.029	1.006-1.052	0.013*
APACHE IV	1.034	1.023-1.044	<0.001*
SOFA	2.117	1.820-2.462	<0.001*
Mechanical ventilation	2.626	2.264-3.538	<0.001*

**Table 8:** Multivariate logistic regression of vaccination and ICU mortality ICU: intensive care unit, APACHE: Acute Physiology and Chronic Health Evaluation, SOFA: Sequential Organ Failure Assessment, aOR: adjusted odds ratio, CI: confidence interval

Parameters	aOR	95% CI	P value
Adjusted with APACHE IV	0.540	0.290-1.006	0.052
Adjusted with SOFA	0.463	0.151-1.422	0.179

## Discussion

In this study, a cohort of critically ill patients with COVID-19 infection was analyzed for their vaccination status. The demographics of this cohort were similar to other published studies of COVID-19 [12]. The majority of the cohort was vaccinated against COVID-19 infection. Our data primarily showed that vaccination did not prevent infection due to SARS-CoV-2 but significantly impacted disease severity and mortality.

Studies comparing outcomes of vaccinated and unvaccinated critically ill patients from developing countries are limited. Most of the vaccination outcome studies for the development of symptomatic infection are conducted in the general population or in a selected group like healthcare workers [13]. These studies have largely shown less incidence of symptomatic infection and hospitalization in the vaccinated (partial or full) population when compared to the unvaccinated population [14]. In a study conducted among 28,342 vaccinated healthcare workers in India, 1,438 (5.07%) became symptomatic. Among the symptomatic vaccinated patients, 18.70% developed symptoms after one dose, 4.79% within two weeks of the second dose, and 76.49% after two weeks of the second dose of vaccination [15]. Symptomatic patients requiring hospital admission and their severity and outcome were not reported in the study. A report investigated hospitalized COVID-19 patients and their vaccination status and observed that the number of hospitalized COVID-19 patients (n=99,445) who were unvaccinated was 92.7% and those who were hospitalized after one dose of vaccination was 6.88% [16]. A study in India evaluated the vaccination status of hospitalized patients with COVID-19 infection and showed that vaccination significantly reduced the need for hospitalization, ICU admission, and mortality [17]. However, this study did not focus on and analyze all consecutive patients admitted to the ICU with COVID-19 infection.

In a study conducted in Israel, 152 fully vaccinated (>7 days after the second dose) hospitalized patients were analyzed. Hospital mortality was noted in 22% of this population, which was mostly attributed to advanced age (74.7±10.5 years), immunosuppression (40%), and the presence of multiple comorbidities [18]. In a recently published community-based study conducted in Chile, it was noted that complete vaccination could prevent hospitalization in 87.5%, ICU admission in 90.3%, and COVID-19-related deaths in 86.3% among persons who were fully immunized [19]. Our study findings cannot be extrapolated to decide on vaccine efficacy, which can be done by a community-based cohort study.

The strength of our study is that it addresses a specific subset of patients who were severe enough to require ICU admission. Our study further explores this group of admitted vaccinated patients and compared them to unvaccinated patients. In most of the other studies, vaccination efficacy was determined by symptomatic infection after one or two doses of vaccine, of which only a minority got admitted to the hospital or required ICU admission.

The contribution of our study to the present literature is to fill the knowledge gap of outcomes of patients admitted to the ICU with severe COVID-19 infection. Like a few others, we have shown that the incidence of ICU admission was not negligible even with prior vaccination against COVID-19. Nevertheless, vaccination has a significant impact on crude mortality, and a strong inverse relationship was observed when adjusted for disease severity.

The limitation of our study is the relatively smaller sample size of unvaccinated than vaccinated patients and it being a single-center study. Our study encompasses a period when multiple strains of SARS-CoV-2, including delta and omicron variants, were circulating in the population. Since it was a retrospective study, viral genotyping could not be done. Moreover, there could be some element of recall bias as symptoms were documented after discharge in some patients. We also did not analyze separately the outcomes of the two adenovirus vector vaccines available (Covishield and Covaxin) at the time of the study.

## Conclusions

We can conclude from our findings that a significant majority of patients in the ICU were admitted with a breakthrough infection, which infers that vaccination was not protective against infection from all variants. However, disease severity, ICU and hospital mortality, and the need for mechanical ventilation were found to be significantly lower among vaccinated patients.
